# PD-L1-Mediated Immunosuppression in Oral Squamous Cell Carcinoma: Relationship With Macrophage Infiltration and Epithelial to Mesenchymal Transition Markers

**DOI:** 10.3389/fimmu.2021.693881

**Published:** 2021-09-06

**Authors:** Tiantian Wu, Caijin Tang, Renchuan Tao, Xiangzhi Yong, Qiaozhi Jiang, Cong Feng

**Affiliations:** ^1^Department of Periodontics and Oral Medicine, College of Stomatology, Guangxi Medical University, Nanning, China; ^2^Guangxi Health Commission Key Laboratory of Prevention and Treatment for Oral Infectious Diseases, Nanning, China; ^3^Guangxi Key Laboratory of Oral and Maxillofacial Rehabilitation and Reconstruction, Guangxi Universities and Colleges Key Laboratory of Oral and Maxillofacial Surgery Disease Treatment, Guangxi Clinical Research Center for Craniofacial Deformity, Nanning, China; ^4^Guangxi Key Laboratory of AIDS Prevention and Treatment, Guangxi Medical University, Nanning, China

**Keywords:** PD-L1, immunosuppressive tumor microenvironment, macrophages, epithelial-mesenchymal transition, chemical drugs

## Abstract

To date, immune check-point inhibitors (ICIs), particularly inhibitors of programmed cell death-1 (PD-1) and PD ligand-1 (PD-L1) have become prominent in cancer treatment and also improved life expectancy of cancer patients. As key regulators of PD-1/PD-L1 axis, the recruitment of tumor-associated macrophages (TAMs) enhances aggressive and invasive properties of tumors in immunosuppressive tumor microenvironment (TME) and promotes epithelial-mesenchymal transition (EMT). The aims of the study were first to characterize the critical links among PD-L1, TME and EMT process and, further, to explore the sensitivity of different chemical agents to different PD-L1 expression groups. Bioinformatical analysis revealed that PD-L1 was highly expressed in OSCC and higher PD-L1 expression correlated with worse survival in patients. Notably, PD-L1 was positively correlated with macrophages infiltration and EMT markers gene expression. Moreover, patients in the PD-L1^high^ group were at a significant chance of benefiting from ICI treatment and they also showed higher sensitivity to the chemical drugs (olaparib, paclitaxel, docetaxel, and pazopanib). These findings implicate PD-L1 could serve as a novel target for prognostic and therapeutic approaches in OSCC patients; PD-L1-mediated immune evasion might be attributable to the infiltration of macrophages, resulting EMT progress; Chemical agents in combination with PD-L1 inhibitor could be served as personalized treatment plan for OSCC patients so as to maximize patient benefit.

## Introduction

Oral squamous cell carcinoma (OSCC), as one of the 10 most frequent cancers with approximately 300,000 new cases diagnosed annually is perceived as an immunosuppressive cancer (1). Despite advances in therapeutic approaches, including surgical methods and radiotherapy, patients with locally advanced or metastatic OSCC still face the risk of a poor prognosis (2). Therefore, further efforts are still demanded to identify clinically relevant biomarkers, establish effective mechanism-based combinations, and develop effective targeted therapies for OSCC.

Cancer immunotherapy, including immune check-point inhibitors (ICIs), Oncolytic virotherapy (OVT), and chimeric antigen receptor (CAR) T cells has led to major improvements in tumor treatment over the past two decades (3). ICIs expressed on the cell surface, including programmed cell death-1 (PD-1) and PD ligand-1 (PD-L1), play crucial roles in activating negative regulatory pathways and evading immune surveillance (4). Upon activation, ICIs can dampen antitumor immune responses, leading to cancer cells escaping from host immune system (5). The interaction between these ligands can be blocked by ICIs, thereby reactivating the cytocidal immune response. Unprecedented advances in tumor control have been made using therapeutic monoclonal antibodies (mAbs) to block ICIs. Particularly, mAbs targeting PD-1/PD-L1 have brought great clinical benefits in multiple indications, either as monotherapy or in combination regimens (6–8). Pembrolizumab and nivolumab as anti-PD-1 mAbs have shown remarkable anti-tumor activity in the treatment of head and neck squamous cell carcinoma (HNSCC), leading to their regulatory approval (9–11). The PD-1/PD-L1 interaction inhibits antitumor activity of cytotoxic lymphocytes (CTLs) (12), which contributes to multiple Suppressive effects, such as immune escape, tumor proliferation, invasion, angiogenesis, and epithelial-mesenchymal transition (EMT) (13). At present, the links among PD-L1, TME, and EMT process in OSCC are not well understood, and the expectation that immunotherapy in combination with standard-therapy can maximize patient benefit necessitates further research on PD-L1-mediated immunosuppression. The present study was aimed at characterizing the critical links among PD-L1, TME and EMT process in OSCC, and further exploring the sensitivity of ICI treatment and different chemical agents to different PD-L1 expression groups.

## Methods And Materials

### Data Acquisition

All Clinical and sequencing data were obtained from the Cancer Genome Atlas (TCGA) and the Gene Expression omnibus (GEO) research network. Transcriptome data of 150 OSCC tissue samples and 30 normal oral tissue samples were extracted from TCGA. Patients’ information on age, tumor-node-metastasis (TNM), stage, survival time and status were organized (**Supplementary Table 1**). The GEO database was used to obtain the OSCC microarray data set GSE30784, and 167 OSCC cancer patient samples were selected as the validation set based on the sample information (14). The EMT related genes were extracted from the Molecular Signature Database (MSigDB) (15).

### Bioinformatic Analysis

Differentially expressed genes (DEGs) in different groups were identified using EdgeR package and filtered by | log2 (Fold Change) | > 1 and adjusted *P* value < 0.05 which was adjusted using the Benjamini-Hochberg (BH) approach (16). Furthermore, the enrichment analysis including Gene ontology (GO) function and Kyoto Encyclopedia of Genes and Genomes (KEGG) pathway annotation was conducted by cluster Profiler package. The PD-L1 expression level in diverse cancer types and correlation between PD-L1 and immune infiltrates were conducted using Tumor Immune Estimation Resource (TIMER) database (17). Gene marker sets of immune cell types were obtained from Bindea et al. (18) and Newman et al. (19). Single-sample Gene Set Enrichment Analysis (ssGSEA) and relative abundance of immune cells were calculated by Gene Set Variation Analysis (GSVA) (20). The classical chemokines and markers of macrophages and EMT signaling pathway were also included (21–24). Tumor Immune Dysfunction and Exclusion (TIDE) algorithm was applied to get individual immunotherapy response (25, 26), and individual chemotherapeutic response was predicted based on Genomics of Drug Sensitivity in Cancer (GDSC) database (27, 28). Drug sensitivity prediction was perform using R package ‘pRRophetic’ (29).

### Statistical Analysis

R statistical language and SPSS 22.0 software were used for statistical analyses. Expression differences among different tumor grade groups were compared using one-way analysis of variance (ANOVA) test. Overall survival (OS) distribution and survival curves were performed by R package survival. The optimal cutoff point for PD-L1 expression level was determined by the ‘surv_cutpoint’ function of ‘survminer’ R package and calculated utilizing the maximally selected rank statistics that calculated the most optimal cut-off for continuous variables using log-rank statistics. Independent prognostic factors were evaluated using univariate and multivariate Cox proportional hazards regression analyses, and relationships between variables were calculated using Pearson correlation coefficients. Differences of TIDE scores and chemotherapy responses between groups were analyzed using Wilcoxon rank sum test.

## Results

### PD-L1 Was Highly Expressed in OSCC and Predicted Poor OS

**Figure 1:**
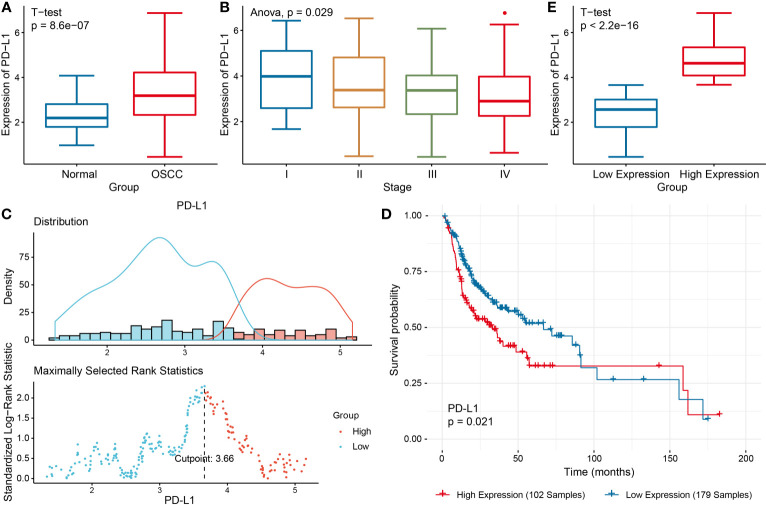
Comprehensive analysis of PD-L1 in OSCC. **(A)** PD-L1 expression in OSCC group and the control group. **(B)** PD-L1 expression in different clinical stages of OSCC. **(C)** The optimal cutoff threshold value **(D)** Kaplan–Meier survival analysis according to the optimal cutoff value. **(E)** PD-L1 expression level in different OSCC groups.

PD-L1 was differentially expressed in different tumor types and was highly expressed in HNSCC (**Supplementary Figure 1A**). TIMER analysis revealed that PD-L1 had positive correlations with various types of immune cells in HNSCC (**Supplementary Figure 1B**). Similarly, in OSCC, PD-L1 was also highly expressed compared with control group, but it gradually decreased with the progress of tumor stages (*P* < 0.05, **Figures 1A, B**). Prognostic value of PD-L1 in OSCC patients was further analyzed; Kaplan–Meier analysis of OS revealed that patients with high PD-L1 expression exhibited a shorter survival time (*P* < 0.05, **Figures 1C–E**); The cutoff point for PD-L1 was 3.66. Cox regression analyses revealed that PD-L1 upregulation was significantly associated with a poor OS (**Table 1**).

**Table 1:** Prognostic value of PD-L1 expression in OSCC.

Type	Univariate Cox Analysis			Multivariate Cox Analysis		
	P values	HR	95%CI	P values	HR	95%CI
Age	0.289998	1.205949	0.852479 - 1.705982	0.341135	1.223076	0.807963 - 1.851465
T	0.00025	1.413316	1.174466 - 1.700741	0.031705	1.433184	1.032024 - 1.99028
N	0.000497	1.46845	1.18289 - 1.822947	0.063043	1.320594	0.984993 - 1.770538
Stage	0.000488	1.470667	1.184029 - 1.826695	0.834734	1.053163	0.647391 - 1.713264
Grade	0.053395	1.307115	0.996091- 1.715256	0.135115	1.294857	0.922618 - 1.81728
PD-L1	0.02162	1.502635	1.061568 - 2.126959	0.01391	1.66808	1.109532 - 2.507807

### Functional Enrichment Analysis of DEGs

**Figure 2:**
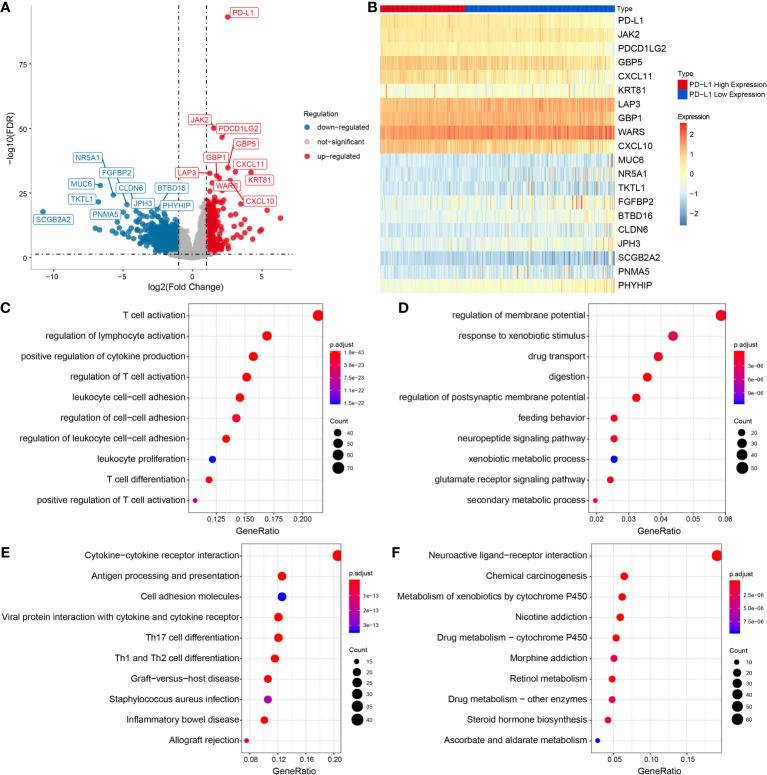
Functional enrichment analysis of DEGs in PD-L1high and PD-L1low groups. **(A)** Volcanic map of DEGs. **(B)** Heat map showing top DEGs. Top 10 enriched GO terms in PD-L1 high **(C)** and PD-L1low group **(D)**. Top 10 significantly enriched KEGG pathways in PD-L1 high **(E)** and PD-L1low group **(F)**.

Differential expression analysis was performed to identify the DEGs between two groups, so as to further study the function of these genes (**Figure 2A**). Among these DEGs, 384 genes were up-regulated in PD-L1^high^ group, and 1090 genes up-regulated in PD-L1^low^ group (**Figure 2A**).

Additionally, tumor-associated macrophages (TAMs) characteristic cytokines such as CXCL10 and CXCL11 were significantly differentially expressed (**Figures 2A, B**). Notably, the GO enrichment analysis indicated 745 immune-related GO terms including lymphocyte activation, activated leukocyte adhesion, and effector cytokine production were significantly enriched in PD-L1^high^ group, while 414 GO terms including digestion, regulation of postsynaptic membrane potential, drug transport, glutamate receptor signaling pathway, regulation of membrane potential were significantly enriched in PD-L1^low^ group (**Figures 2C, D**). KEGG pathway analysis showed that 47 KEGG pathways were characteristic enriched in PD-L1^high^ group, and 23 KEGG pathways were characteristic enriched in PD-L1^low^ group (**Figures 2E, F**).

### PD-L1 Was Correlated With Macrophage Infiltration and Macrophage-Derived Chemokines

**Figure 3:**
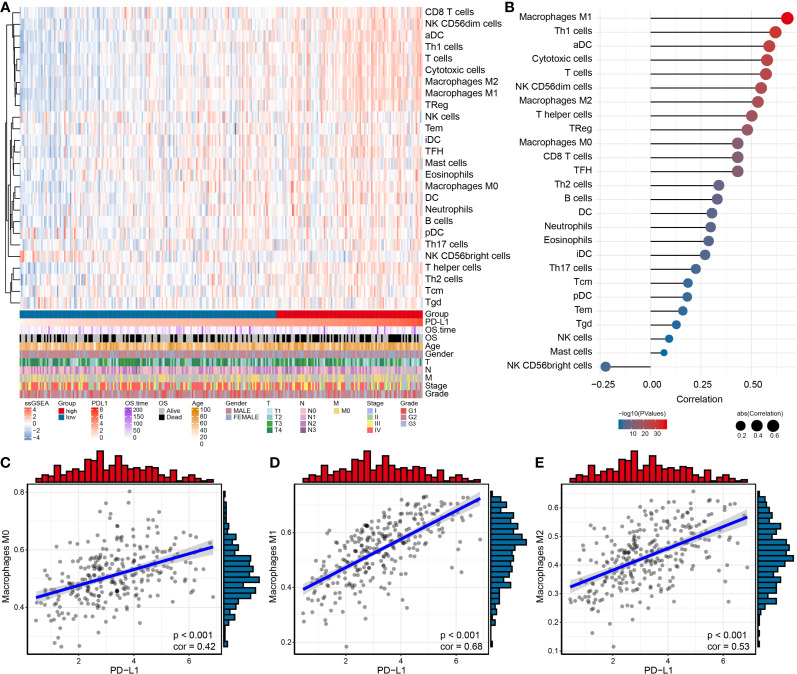
Relationship between immune cell infiltration and PD-L1 expression in OSCC samples. **(A)** Heat map by using the ssGSEA scores from 26 immune cell types. **(B)** Correlation between PD-L1 and immune infiltrates in OSCC samples. Correlation between PD-L1 and M0 **(C)**, M1 **(D)** and M2 **(E)** macrophages infiltration in OSCC samples.

The results of GSVA revealed that PD-L1 was positively associated with cell proliferation of 12 immune cell types in OSCC (**Supplementary Figure 2** and **Figures 3A, B**), especially with M0 macrophages (cor = 0.42, *P* < 0.001), M1 macrophages (cor = 0.68, *P* < 0.001), and M2 macrophages (cor = 0.53, *P* < 0.001) (**Figures 3C** **–E**). In addition, the results of ssGSEA in the GEO OSCC validation set showed PD-L1 was strongly positively associated with cell proliferation in several types of immune cell infiltration (**Supplementary Figures 3A–H**), especially with M1 macrophages (cor = 0.63, *P* < 0.001, **Supplementary Figure 3D**) and M2 macrophages (cor = 0.54, *P* < 0.001, **Supplementary Figure 3E**).

**Figure 4:**
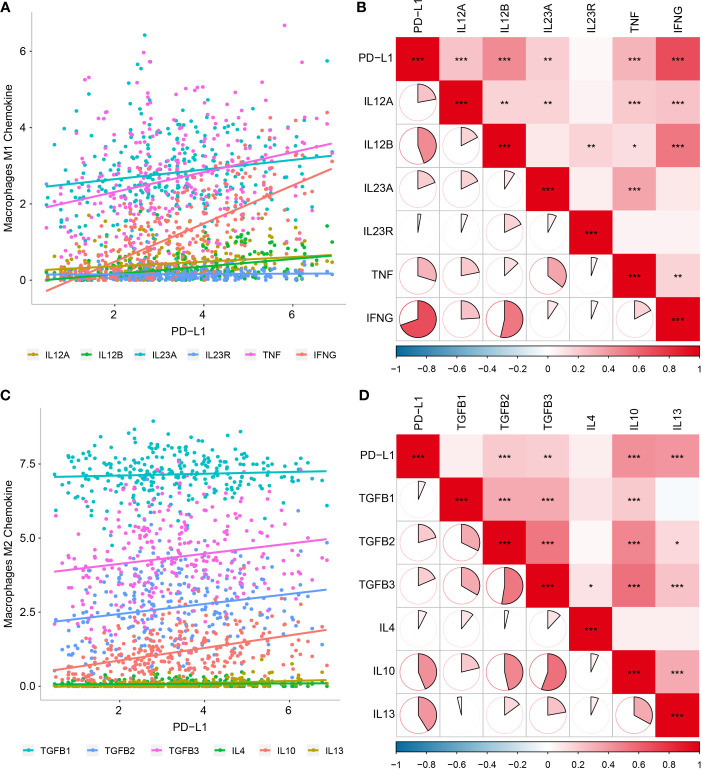
Relationship between PD-L1 and macrophage-derived chemokines in OSCC samples. **(A)** Scatter plot showing correlation between PD-L1 and M1-derived chemokines. **(B)** Correlation between PD-L1 and M1-derived chemokines. **(C)** Scatter plot showing correlation between PD-L1 and M2-derived chemokines. **(D)** Correlation between PD-L1 and M2-derived chemokines. *P < 0.05; **P < 0.01; ***P < 0.001.

To validate the above results, we further analyzed correlations between PD-L1 expression and macrophage markers, which revealed that M1-related chemokines (IL12A, IL-12B, IL-23A, TNF, and IFNG) and M2-related chemokines (TGFBs, IL-10, and IL-13) showed the strongest positive correlations with PD-L1 expression (*P* < 0.05, **Figures 4** **A–D**). We also observed statistically significant correlations between M1-related chemokines and M2-related chemokines (**Figures 4** **B, D**).

### Associations Between PD-L1 Expression, Macrophage Infiltration and EMT Biomarkers

**Figure 5:**
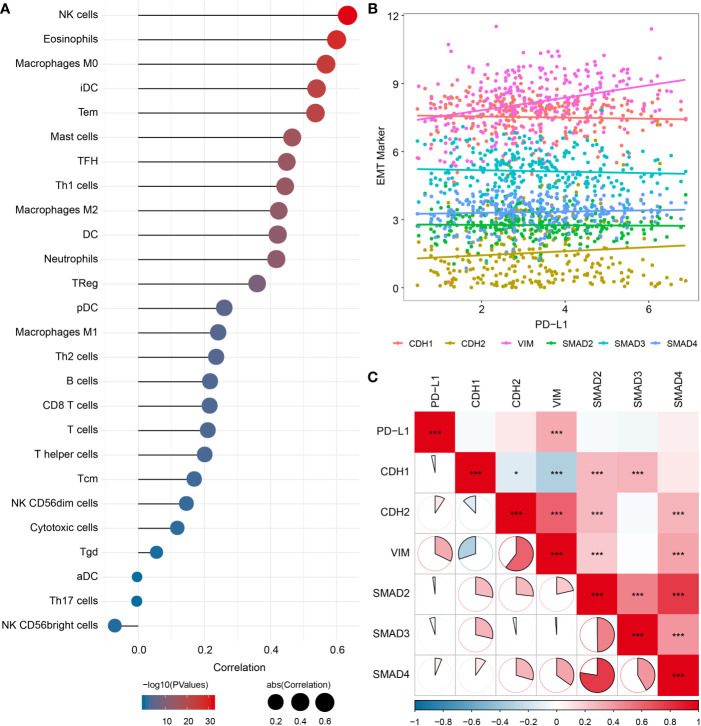
Relationship among PD-L1, immune infiltrates and EMT in OSCC samples. **(A)** Correlation between EMT pathway enrichment scores and immune cell infiltration. **(B)** Scatter plot showing correlation between PD-L1 and EMT-related genes. **(C)** Correlation between PD-L1and EMT-related genes. *P < 0.05; ***P < 0.001.

Since the EMT process has been considered to be particularly relevant to TME, we analyzed the potential association of EMT with immune infiltration, and found significant correlations between EMT biomarkers and various immune cells infiltration (**Figure 5A**), especially with M0 macrophages (cor = 0.568, *P* < 0.001) and M2 macrophages (cor = 0.425, *P* < 0.001). We also evaluated the association between PD-L1 and EMT biomarkers, which showed significant correlations between PD-L1 and vimentin (VIM) (cor = 0.322, *P* < 0.001) (**Figures 5B, C**).

### Sensitivity Differences to Immunotherapy/Chemotherapy Between Groups

**Figure 6:**
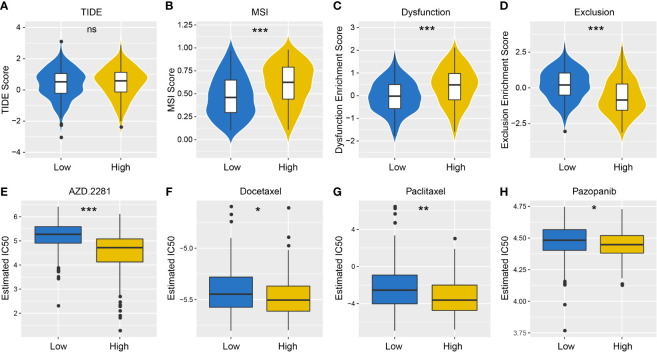
Differences in the sensitivity of immunotherapy and chemotherapy between different PD-L1 expression groups. TIDE **(A)**, MSI **(B)**, T cell Dysfunction **(C)** and T cell Exclusion **(D)** scores in PD-L1^high^ and PD-L1^low^ Groups (NS: not significant; *P < 0.05; **P < 0.01; ***P < 0.001). Sensitivity to olaparib (AZD. 2281) **(E)**, docetaxel **(F)**, paclitaxel **(G)** and pazopanib **(H)** in PD-L1^high^ and PD-L1^low^ Groups (NS: not significant; *P < 0.05; **P < 0.01; ***P < 0.001).

TIDE algorithm was employed to assess individual immunotherapy response in different PD-L1 expression groups, and higher TIDE prediction score represented a higher immune evasion potential (**Figure 6A**). The results revealed patients with higher PD-L1 expression had a higher microsatellite instability (MSI) score and T cell dysfunction score (**Figures 6B, C**), but a lower T cell exclusion score (**Figure 6D**), indicating that these patients were less likely to benefit from ICI treatment.

We also took into account the differences in the response of chemotherapy in OSCC patients, and evaluated sensitivity of two patient groups to four chemical drugs (olaparib, docetaxel, paclitaxel, and pazopanib). The IC50 value of each sample in OSCC was estimated, and significant differences were observed between groups, which revealed that PD-L1^high^ group showed higher sensitivity to all drugs (**Figures 6E–H**, *P* < 0.001).

## Discussion

PD-L1 overexpression has been demonstrated in many common cancers, inducing T-cell tolerance and promoting immune escape. Blockades targeted on PD‐1/PD‐L1 have already shown striking effectiveness in clinical applications (6–8). Immune characteristics relevant to PD-L1 in TME and EMT process during cancer progression were depicted in our study. Firstly, PD-L1 expression was highly expressed in OSCC, but it gradually decreased with the progress of tumor stages. As an immunosuppressive cell surface molecule that promotes T cell depletion, PD-L1 upregulation may link with increased cancer aggression and poorer prognosis, as proposed in several previous studies (29, 30). In contrast to this expectation, we observed an decrease in PD-L1 expression as disease progressed; Some previous studies on the association between PD-L1 expression and improved prognosis also Supported our findings (31–34). The most likely explanation for this paradox is that PD-L1 expression can be induced by cytokines, primarily the production of interferon-γ within TME, and therefore, its expression actually reflects the contribution of endogenous anti-tumor immune response, which typically occurred in the early stages of tumor development and progression (35, 36). Moreover, our results also showed that PD-L1 was positively associated with cell proliferation in activated TAMs and EMT process. The DEGs in PD-L1^high^ group were significantly enriched in canonical signaling pathways that related to regulation of lymphocyte activation, suggesting the critical involvement of PD-L1 in regulating TAMs function. Additionally, our results suggested that patients in the PD-L1^high^ group were at a significant chance of benefiting from ICI treatment and they also showed higher sensitivity to the chemical drugs (olaparib, paclitaxel, docetaxel, and pazopanib). This study provided preliminary evidence regarding the tight correlations among PD-L1, TAMs and EMT process, further supporting the notion that clinical efficacy of both chemotherapy and immunotherapy could be greatly improved by utilizing PD-L1 inhibitors in OSCC.

A previous research on PD-L1 regulating the proliferation of macrophages revealed that the phenotype and function of macrophages could be altered by anti-PD-L1 treatment, suggesting a crucial role of PD-L1 in regulating macrophages activation and function (37). Circulating monocytes can be recruited into TME and further polarize into TAMs. Classically, in response to microenvironmental stimuli, TAMs polarize to M1-like phenotype exhibiting proinflammatory and tumor-inhibiting phenotypic effects, while certain cytokines convert TAMs into an M2-like phenotype with anti-inflammatory but tumor-promoting functions (38, 39). Previous studies have shown that M2 macrophages elevated PD*-*L1 expression in cancer cells and at the same time facilitating immune escape (40, 41). As for M1 macrophages, the PD-L1 expressed on the surface was also reported to result in immune escape of cancer cells, resulting in bidirectional effects of both anti-tumoral and pro-tumoral activities (42): On one hand, M1-like phenotype had the unique ability to promote the activation and recruitment of various immune effectors, performing surveillance tasks (43), and their infiltration indicated good prognosis in some cancers (44). On the other hand, M1 macrophages involved in cancer phenotype maintenance and tumorigenicity regulation *in vivo*. For example, previous studies confirmed the important protumorigenic factor role of M1 macrophages in urethane-induced lung tumorigenesis (45); Higher aggregation level of human leukocyte antigen-DR+ (HLA-DR+) M1-like TAMs was related to poor response to ionizing radiotherapy in rectal cancers patients (46). In the present study, positive association between PD-L1 expression and TAMs infiltration (both M0, M1 and M2) was observed in OSCC, which was in consistent with previous findings. These findings suggested that TAMs may contribute to pro-tumorigenic effects by promoting PD-L1 expression in OSCC.

EMT is a novel mechanism involved in cancer metastasis, by which epithelial cells acquire both mesenchymal and epithelial phenotypes for cell migration and proliferation. The type III intermediate filament protein VIM that constitutes a key cytoskeletal element of mesenchymal cells, is a canonical marker of EMT process, and EMT process is characterized by marked VIM upregulation (47, 48). It is generally accepted that epithelial cells undergoing EMT are able to survive better under adverse environmental conditions, which enables tumor cells to evade immune destruction (49, 50). In addition, accumulating evidences have suggested strong correlations between EMT and immune evasion by activating multiple ICIs (51, 52); Cancer cells with PD-L1 upregulation displays an EMT phenotype that aids in immune escape. Macrophages and cancer cells were reported to establish a two-way cross-talk: Macrophages facilitated EMT changes in the latter while the latter skewed TAMs polarization into an M2 phenotype (53). Interestingly, recent evidences have indicated that in addition to M2 phenotype, M1-like macrophages also promoted EMT and chemoresistance (54); TAMs generally share both M1- and M2-like phenotypes instead of being strictly classified into above two phenotypes (55), which does not rule out the possibility that these two phenotypes are exchangeable (56, 57).

Immuno-oncology revolutionized cancer treatment. Pembrolizumab and nivolumab as anti-PD-1 mAbs have shown remarkable anti-tumor activity in the treatment of patients with recurrent/metastatic HNSCC. Ferris et al. (10) experienced nivolumab in a population of 347 HNSCC patients to evaluate the efficacy of nivolumab comparable to that of single-agent chemotherapy (CheckMate 141): Both ORR (13.3% *vs* 5.8%) and median OS (7.5 *vs* 5.1 months) were significantly improved in the nivolumab group. The estimated 12-month OS in nivolumab group was 36% *versus* 16.6% in standard-therapy group, while there was little difference in median progression-free survival (PFS) of these two groups. Similarly, the KEYNOTE-040 phase III study also compared the clinical efficacy of pembrolizumab *versus* current standard-therapy with a total of 495 patients enrolled: Both ORR (14.6% *vs* 10.1%) and median OS (8.4 *vs* 6.9 months) were significantly improved in the pembrolizumab group (11). These randomized phase III trials indicated that survival benefit could be well conferred by immunotherapy. Thus, it is of great importance to perform immune monitoring on patients, thereby identifying potential biomarkers, accurately stratifying patients and delineating responders and non-responders. Olaparib (AZD. 2281), a competitive inhibitor of poly (ADP-ribose) polymerase-1 (PARP-1), has been used in the clinical treatment of ovarian cancer with BRCA1/2 gene mutations (58, 59). An ongoing phase 1/2 study of olaparib and the PD-L1 inhibitor durvalumab in breast cancer patients with BRCA1/2 mutations demonstrated that 24 of 30 patients who were eligible for trial entry by study design achieved durable and adaptable cancer control at 12 weeks of combination therapy (60). Olaparib in combination with durvalumab exhibited promising anti‐tumor efficacy and safety, which was confirmed in numerous clinical studies (61–63). In addition, paclitaxel, including nanoparticle albumin‐bound paclitaxel (nab-paclitaxel), is a widely used chemotherapy drug for various cancers; Combination therapy of anti-PD-L1 mAb atezolizumab and nab-paclitaxel as a first-line treatment exhibited significantly improved PFS in patients with PD-L1-positive tumors (64, 65). In this study, we analyzed the sensitivity differences of immunotherapy and chemotherapy between different PD-L1 expression groups with the expectation of screening potential benefit populations and achieving enhanced efficacy. The results revealed that patients in the PD-L1^high^ groups were at a significant chance of benefiting from ICI treatment and they also showed higher sensitivity to the four chemical drugs (olaparib, paclitaxel, docetaxel, and pazopanib. Further research is needed to fully confirm the promising efficacy of these agents in combination with PD-L1 inhibitor in improving personalized treatment for OSCC patients. By screening the subgroups of potential beneficiaries, immunotherapy and chemotherapy can harnessed to maximize the immunostimulatory effects of therapeutic agents.

## Data Availability Statement

The original contributions presented in the study are included in the article/**Supplementary Material**. Further inquiries can be directed to the corresponding author.

## Author Contributions

Conceived and designed the study: TW, CT, and RT. Data collection and analysis: TW and RT. Writing and revising the manuscript: TW, CT, XY, QJ, CF, and RT. All authors contributed to the article and approved the submitted version.

## Funding

This study was funded by Guangxi Medical High-level Talents Training Program, National Natural Science Foundation of China (NO. 81771073), State Key Laboratory of Oral Diseases Open Fund (NO. SKLOD2019OF01), and Innovation Project of Guangxi Graduate Education (NO. YCSW2019105 and NO. YCBZ2021051).

## Conflict of Interest

The authors declare that the research was conducted in the absence of any commercial or financial relationships that could be construed as a potential conflict of interest.

## Publisher’s Note

All claims expressed in this article are solely those of the authors and do not necessarily represent those of their affiliated organizations, or those of the publisher, the editors and the reviewers. Any product that may be evaluated in this article, or claim that may be made by its manufacturer, is not guaranteed or endorsed by the publisher.

## Supplementary Material

Supplementary Figure 1 PD-L1 expression level in various cancers. **(A)** PD-L1 expression level in various cancers was analyzed by box-plot. **(B)** Correlation between PD-L1 and immune infiltrates in HNSCC.

Supplementary Figure 2 Scatter diagram of the correlation between PD-L1 expression and immune cell infiltration in OSCC.

Supplementary Figure 3 Relationship between PD-L1 and immune infiltrates in the GEO validation set. **(A)** Heat map of OSCC samples by using the ssGSEA scores from 26 immune cell types. **(B)** PD-L1 significantly associated with Immune cells. Scatter plot showing correlation between PD-L1 and various immune cells, including aDC **(C)**, M1 macrophages **(D)**, M2 macrophages **(E)**, Cytotoxic cells **(F)**, T cells **(G)** and Th1 cells **(H)**.

Supplementary Table 1 The Clinicopathological data of patients.

## References

[1] Seiwert TY Zuo Z Keck MK Khattri A Pedamallu CS Stricker T. Integrative and Comparative Genomic Analysis of HPV-Positive and HPV-Negative Head and Neck Squamous Cell Carcinomas. Clin Cancer Res (2015) 21(3):632–41. 10.1158/1078-0432.CCR-13-3310 PMC4305034 25056374

[2] Cohen EE Davis DW Karrison TG Seiwert TY Wong SJ Nattam S. Erlotinib and Bevacizumab in Patients With Recurrent or Metastatic Squamous-Cell Carcinoma of the Head and Neck: A Phase I/II Study. Lancet Oncol (2009) 10(3):247–57. 10.1016/S1470-2045(09)70002-6 PMC2768532 19201650

[3] Dougan M Dranoff G. Immune Therapy for Cancer. Annu Rev Immunol (2009) 27:83–117. 10.1146/annurev.immunol.021908.132544 19007331

[4] Pardoll DM. The Blockade of Immune Checkpoints in Cancer Immunotherapy. Nat Rev Cancer (2012) 12(4):252–64. 10.1038/nrc3239 PMC4856023 22437870

[5] Dong H Strome SE Salomao DR Tamura H Hirano F Flies DB. Tumor-Associated B7-H1 Promotes T-Cell Apoptosis: A Potential Mechanism of Immune Evasion. Nat Med (2002) 8(8):793–800. 10.1038/nm730 12091876

[6] Okazaki T Chikuma S Iwai Y Fagarasan S Honjo T. A Rheostat for Immune Responses: The Unique Properties of PD-1 and Their Advantages for Clinical Application. Nat Immunol (2013) 14(12):1212–8. 10.1038/ni.2762 24240160

[7] Sharma P Allison JP. Immune Checkpoint Targeting in Cancer Therapy: Toward Combination Strategies With Curative Potential. Cell (2015) 161(2):205–14. 10.1016/j.cell.2015.03.030 PMC5905674 25860605

[8] Sharma P Allison JP. The Future of Immune Checkpoint Therapy. Science (2015) 348(6230):56–61. 10.1126/science.aaa8172 25838373

[9] Seiwert TY Burtness B Mehra R Weiss J Berger R Eder JP. Safety and Clinical Activity of Pembrolizumab for Treatment of Recurrent or Metastatic Squamous Cell Carcinoma of the Head and Neck (KEYNOTE-012): An Open-Label, Multicentre, Phase 1b Trial. Lancet Oncol (2016) 17(7):956–65. 10.1016/s1470-2045(16)30066-3 27247226

[10] Ferris RL Blumenschein G Jr. Fayette J Guigay J Colevas AD Licitra L. Nivolumab for Recurrent Squamous-Cell Carcinoma of the Head and Neck. N Engl J Med (2016) 375(19):1856–67. 10.1056/NEJMoa1602252 PMC5564292 27718784

[11] Cohen EEW Soulières D Le Tourneau C Dinis J Licitra L Ahn MJ. Pembrolizumab *Versus* Methotrexate, Docetaxel, or Cetuximab for Recurrent or Metastatic Head-and-Neck Squamous Cell Carcinoma (KEYNOTE-040): A Randomised, Open-Label, Phase 3 Study. Lancet (2019) 393(10167):156–67. 10.1016/s0140-6736(18)31999-8 30509740

[12] Raimondi C Carpino G Nicolazzo C Gradilone A Gianni W Gelibter A. PD-L1 and Epithelial-Mesenchymal Transition in Circulating Tumor Cells From Non-Small Cell Lung Cancer Patients: A Molecular Shield to Evade Immune System? Oncoimmunology (2017) 6(12):e1315488. 10.1080/2162402X.2017.1315488 29209560 PMC5706610

[13] Zou W Wolchok JD Chen L. PD-L1 (B7-H1) and PD-1 Pathway Blockade for Cancer Therapy: Mechanisms, Response Biomarkers, and Combinations. Sci Transl Med (2016) 8(328):328rv4. 10.1126/scitranslmed.aad7118 PMC4859220 26936508

[14] Chen C Méndez E Houck J Fan W Lohavanichbutr P Doody D. Gene Expression Profiling Identifies Genes Predictive of Oral Squamous Cell Carcinoma. Cancer Epidemiol Biomarkers Prev (2008) 17(8):2152–62. 10.1158/1055-9965.Epi-07-2893 PMC2575803 18669583

[15] Hanahan D Weinberg RA. Hallmarks of Cancer: The Next Generation. Cell (2011) 144(5):646–74. 10.1016/j.cell.2011.02.013 21376230

[16] Duggal P Gillanders EM Holmes TN Bailey-Wilson JE. Establishing an Adjusted P-Value Threshold to Control the Family-Wide Type 1 Error in Genome Wide Association Studies. BMC Genomics (2008) 9:516. 10.1186/1471-2164-9-516 18976480 PMC2621212

[17] Li T Fan J Wang B Traugh N Chen Q Liu JS. TIMER: A Web Server for Comprehensive Analysis of Tumor-Infiltrating Immune Cells. Cancer Res (2017) 77(21):e108–e10. 10.1158/0008-5472.Can-17-0307 PMC6042652 29092952

[18] Bindea G Mlecnik B Tosolini M Kirilovsky A Waldner M Obenauf AC. Spatiotemporal Dynamics of Intratumoral Immune Cells Reveal the Immune Landscape in Human Cancer. Immunity (2013) 39(4):782–95. 10.1016/j.immuni.2013.10.003 24138885

[19] Newman AM Liu CL Green MR Gentles AJ Feng W Xu Y. Robust Enumeration of Cell Subsets From Tissue Expression Profiles. Nat Methods (2015) 12(5):453–7. 10.1038/nmeth.3337 PMC4739640 25822800

[20] Hänzelmann S Castelo R Guinney J. GSVA: Gene Set Variation Analysis for Microarray and RNA-Seq Data. BMC Bioinf (2013) 14:7. 10.1186/1471-2105-14-7 PMC3618321 23323831

[21] Mantovani A Sica A Sozzani S Allavena P Vecchi A Locati M. The Chemokine System in Diverse Forms of Macrophage Activation and Polarization. Trends Immunol (2004) 25(12):677–86. 10.1016/j.it.2004.09.015 15530839

[22] Murray PJ Allen JE Biswas SK Fisher EA Gilroy DW Goerdt S. Macrophage Activation and Polarization: Nomenclature and Experimental Guidelines. Immunity (2014) 41(1):14–20. 10.1016/j.immuni.2014.06.008 25035950 PMC4123412

[23] Li W Graeber MB. The Molecular Profile of Microglia Under the Influence of Glioma. Neuro Oncol (2012) 14(8):958–78. 10.1093/neuonc/nos116 PMC3408253 22573310

[24] Karacosta LG Anchang B Ignatiadis N Kimmey SC Benson JA Shrager JB. Mapping Lung Cancer Epithelial-Mesenchymal Transition States and Trajectories With Single-Cell Resolution. Nat Commun (2019) 10(1):5587. 10.1038/s41467-019-13441-6 31811131 PMC6898514

[25] Liu Z Zhang Y Shi C Zhou X Xu K Jiao D. A Novel Immune Classification Reveals Distinct Immune Escape Mechanism and Genomic Alterations: Implications for Immunotherapy in Hepatocellular Carcinoma. J Transl Med (2021) 19(1):5. 10.1186/s12967-020-02697-y 33407585 PMC7789239

[26] Jiang P Gu S Pan D Fu J Sahu A Hu X. Signatures of T Cell Dysfunction and Exclusion Predict Cancer Immunotherapy Response. Nat Med (2018) 24(10):1550–8. 10.1038/s41591-018-0136-1 PMC6487502 30127393

[27] Yang W Soares J Greninger P Edelman EJ Lightfoot H Forbes S. Genomics of Drug Sensitivity in Cancer (GDSC): A Resource for Therapeutic Biomarker Discovery in Cancer Cells. Nucleic Acids Res (2013) 41(Database issue):D955–61. 10.1093/nar/gks1111 PMC3531057 23180760

[28] Fu J Li K Zhang W Wan C Zhang J Jiang P. Large-Scale Public Data Reuse to Model Immunotherapy Response and Resistance. Genome Med (2020) 12(1):21. 10.1186/s13073-020-0721-z 32102694 PMC7045518

[29] Nduom EK Wei J Yaghi NK Huang N Kong LY Gabrusiewicz K. PD-L1 Expression and Prognostic Impact in Glioblastoma. Neuro Oncol (2016) 18(2):195–205. 10.1093/neuonc/nov172 26323609 PMC4724183

[30] Fanale D Incorvaia L Badalamenti G De Luca I Algeri L Bonasera A. Prognostic Role of Plasma PD-1, PD-L1, Pan-BTN3As and BTN3A1 in Patients Affected by Metastatic Gastrointestinal Stromal Tumors: Can Immune Checkpoints Act as a Sentinel for Short-Term Survival? Cancers (Basel) (2021) 13(9):2118. 10.3390/cancers13092118 33925671 PMC8125172

[31] Bang YJ Ruiz EY Van Cutsem E Lee KW Wyrwicz L Schenker M. Phase III, Randomised Trial of Avelumab *Versus* Physician's Choice of Chemotherapy as Third-Line Treatment of Patients With Advanced Gastric or Gastro-Oesophageal Junction Cancer: Primary Analysis of JAVELIN Gastric 300. Ann Oncol (2018) 29(10):2052–60. 10.1093/annonc/mdy264 PMC6225815 30052729

[32] Taube JM Anders RA Young GD Xu H Sharma R McMiller TL. Colocalization of Inflammatory Response With B7-H1 Expression in Human Melanocytic Lesions Supports an Adaptive Resistance Mechanism of Immune Escape. Sci Transl Med (2012) 4(127):127ra37. 10.1126/scitranslmed.3003689 PMC3568523 22461641

[33] Badoual C Hans S Merillon N Van Ryswick C Ravel P Benhamouda N. PD-1-Expressing Tumor-Infiltrating T Cells are a Favorable Prognostic Biomarker in HPV-Associated Head and Neck Cancer. Cancer Res (2013) 73(1):128–38. 10.1158/0008-5472.Can-12-2606 23135914

[34] Roper E Lum T Palme CE Ashford B Ch'ng S Ranson M. PD-L1 Expression Predicts Longer Disease Free Survival in High Risk Head and Neck Cutaneous Squamous Cell Carcinoma. Pathology (2017) 49(5):499–505. 10.1016/j.pathol.2017.04.004 28666643

[35] Davar D Wang H Chauvin JM Pagliano O Fourcade JJ Ka M. Phase Ib/II Study of Pembrolizumab and Pegylated-Interferon Alfa-2b in Advanced Melanoma. J Clin Oncol (2018) 36(35):JCO1800632. 10.1200/JCO.18.00632 PMC6286160 30359157

[36] Carey CD Gusenleitner D Lipschitz M Roemer MGM Stack EC Gjini E. Topological Analysis Reveals a PD-L1-Associated Microenvironmental Niche for Reed-Sternberg Cells in Hodgkin Lymphoma. Blood (2017) 130(22):2420–30. 10.1182/blood-2017-03-770719 PMC5766840 28893733

[37] Hartley GP Chow L Ammons DT Wheat WH Dow SW. Programmed Cell Death Ligand 1 (PD-L1) Signaling Regulates Macrophage Proliferation and Activation. Cancer Immunol Res (2018) 6(10):1260–73. 10.1158/2326-6066.Cir-17-0537 30012633

[38] Cheng H Wang Z Fu L Xu T. Macrophage Polarization in the Development and Progression of Ovarian Cancers: An Overview. Front Oncol (2019) 9:421. 10.3389/fonc.2019.00421 31192126 PMC6540821

[39] Guadagno E Presta I Maisano D Donato A Pirrone CK Cardillo G. Role of Macrophages in Brain Tumor Growth and Progression. Int J Mol Sci (2018) 19(4):1005. 10.3390/ijms19041005 PMC5979398 29584702

[40] Qian M Ling W Ruan Z. Long non-Coding RNA SNHG12 Promotes Immune Escape of Ovarian Cancer Cells Through Their Crosstalk With M2 Macrophages. Aging (Albany NY) (2020) 12(17):17122–36. 10.18632/aging.103653 PMC7521506 32927431

[41] Kim SH Go SI Song DH Park SW Kim HR Jang I. Prognostic Impact of CD8 and Programmed Death-Ligand 1 Expression in Patients With Resectable Non-Small Cell Lung Cancer. Br J Cancer (2019) 120(5):547–54. 10.1038/s41416-019-0398-5 PMC6461857 30745585

[42] Kuang DM Zhao Q Peng C Xu J Zhang JP Wu C. Activated Monocytes in Peritumoral Stroma of Hepatocellular Carcinoma Foster Immune Privilege and Disease Progression Through PD-L1. J Exp Med (2009) 206(6):1327–37. 10.1084/jem.20082173 PMC2715058 19451266

[43] O'Sullivan T Saddawi-Konefka R Vermi W Koebel CM Arthur C White JM. Cancer Immunoediting by the Innate Immune System in the Absence of Adaptive Immunity. J Exp Med (2012) 209(10):1869–82. 10.1084/jem.20112738 PMC3457735 22927549

[44] Ohri CM Shikotra A Green RH Waller DA Bradding P. Macrophages Within NSCLC Tumour Islets are Predominantly of a Cytotoxic M1 Phenotype Associated With Extended Survival. Eur Respir J (2009) 33(1):118–26. 10.1183/09031936.00065708 19118225

[45] Zaynagetdinov R Sherrill TP Polosukhin VV Han W Ausborn JA McLoed AG. A Critical Role for Macrophages in Promotion of Urethane-Induced Lung Carcinogenesis. J Immunol (2011) 187(11):5703–11. 10.4049/jimmunol.1100558 PMC3221921 22048774

[46] Shaikh S Noshirwani A West N Perry S Jayne D. Can Macrophages Within the Microenvironment of Locally Invasive Rectal Cancers Predict Response to Radiotherapy? Lancet (2015) 385(Supl 1):S87. 10.1016/s0140-6736(15)60402-0 26312909

[47] Pardo-Sánchez JM Mancheño N Cerón J Jordá C Ansotegui E Juan Ó. Increased Tumor Growth Rate and Mesenchymal Properties of NSCLC-Patient-Derived Xenograft Models During Serial Transplantation. Cancers (Basel) (2021) 13(12):2980. 10.3390/cancers13122980 34198671 PMC8232339

[48] Yao JX Chen X Zhu YJ Wang H Hu XY Guo JM. Prognostic Value of Vimentin Is Associated With Immunosuppression in Metastatic Renal Cell Carcinoma. Front Oncol (2020) 10:1181. 10.3389/fonc.2020.01181 32850341 PMC7417332

[49] Friedl P Alexander S. Cancer Invasion and the Microenvironment: Plasticity and Reciprocity. Cell (2011) 147(5):992–1009. 10.1016/j.cell.2011.11.016 22118458

[50] Schreiber RD Old LJ Smyth MJ. Cancer Immunoediting: Integrating Immunity's Roles in Cancer Suppression and Promotion. Science (2011) 331(6024):1565–70. 10.1126/science.1203486 21436444

[51] Chen L Heymach JV Qin FX Gibbons DL. The Mutually Regulatory Loop of Epithelial-Mesenchymal Transition and Immunosuppression in Cancer Progression. Oncoimmunology (2015) 4(5):e1002731. 10.1080/2162402x.2014.1002731 26155392 PMC4485725

[52] Chouaib S Janji B Tittarelli A Eggermont A Thiery JP. Tumor Plasticity Interferes With Anti-Tumor Immunity. Crit Rev Immunol (2014) 34(2):91–102. 10.1615/critrevimmunol.2014010183 24940910

[53] Sommariva M Gagliano N. E-Cadherin in Pancreatic Ductal Adenocarcinoma: A Multifaceted Actor During EMT. Cells (2020) 9(4):1040. 10.3390/cells9041040 PMC7226001 32331358

[54] Kuwada K Kagawa S Yoshida R Sakamoto S Ito A Watanabe M. The Epithelial-to-Mesenchymal Transition Induced by Tumor-Associated Macrophages Confers Chemoresistance in Peritoneally Disseminated Pancreatic Cancer. J Exp Clin Cancer Res (2018) 37(1):307. 10.1186/s13046-018-0981-2 30537992 PMC6288926

[55] Helm O Held-Feindt J Grage-Griebenow E Reiling N Ungefroren H Vogel I. Tumor-Associated Macrophages Exhibit Pro- and Anti-Inflammatory Properties by Which They Impact on Pancreatic Tumorigenesis. Int J Cancer (2014) 135(4):843–61. 10.1002/ijc.28736 24458546

[56] Pathria P Louis TL Varner JA. Targeting Tumor-Associated Macrophages in Cancer. Trends Immunol (2019) 40(4):310–27. 10.1016/j.it.2019.02.003 30890304

[57] Hu W Li X Zhang C Yang Y Jiang J Wu C. Tumor-Associated Macrophages in Cancers. Clin Transl Oncol (2016) 18(3):251–8. 10.1007/s12094-015-1373-0 26264497

[58] Moore K Colombo N Scambia G Kim BG Oaknin A Friedlander M. Maintenance Olaparib in Patients With Newly Diagnosed Advanced Ovarian Cancer. N Engl J Med (2018) 379(26):2495–505. 10.1056/NEJMoa1810858 30345884

[59] Poveda A Floquet A Ledermann JA Asher R Penson RT Oza AM. Olaparib Tablets as Maintenance Therapy in Patients With Platinum-Sensitive Relapsed Ovarian Cancer and a BRCA1/2 Mutation (SOLO2/ENGOT-Ov21): A Final Analysis of a Double-Blind, Randomised, Placebo-Controlled, Phase 3 Trial. Lancet Oncol (2021) 22(5):620–31. 10.1016/S1470-2045(21)00073-5 33743851

[60] Domchek SM Postel-Vinay S Im SA Park YH Delord JP Italiano A. Olaparib and Durvalumab in Patients With Germline BRCA-Mutated Metastatic Breast Cancer (MEDIOLA): An Open-Label, Multicentre, Phase 1/2, Basket Study. Lancet Oncol (2020) 21(9):1155–64. 10.1016/s1470-2045(20)30324-7 32771088

[61] Fumet JD Limagne E Thibaudin M Truntzer C Bertaut A Rederstorff E. Precision Medicine Phase II Study Evaluating the Efficacy of a Double Immunotherapy by Durvalumab and Tremelimumab Combined With Olaparib in Patients With Solid Cancers and Carriers of Homologous Recombination Repair Genes Mutation in Response or Stable After Olaparib Treatment. BMC Cancer (2020) 20(1):748. 10.1186/s12885-020-07253-x 32778095 PMC7418426

[62] Karzai F VanderWeele D Madan RA Owens H Cordes LM Hankin A. Activity of Durvalumab Plus Olaparib in Metastatic Castration-Resistant Prostate Cancer in Men With and Without DNA Damage Repair Mutations. J Immunother Cancer (2018) 6(1):141. 10.1186/s40425-018-0463-2 30514390 PMC6280368

[63] Lampert EJ Zimmer A Padget M Cimino-Mathews A Nair JR Liu Y. Combination of PARP Inhibitor Olaparib, and PD-L1 Inhibitor Durvalumab, in Recurrent Ovarian Cancer: A Proof-Of-Concept Phase II Study. Clin Cancer Res (2020) 26(16):4268–79. 10.1158/1078-0432.Ccr-20-0056 PMC7442720 32398324

[64] Schmid P Rugo HS Adams S Schneeweiss A Barrios CH Iwata H. Atezolizumab Plus Nab-Paclitaxel as First-Line Treatment for Unresectable, Locally Advanced or Metastatic Triple-Negative Breast Cancer (IMpassion130): Updated Efficacy Results From a Randomised, Double-Blind, Placebo-Controlled, Phase 3 Trial. Lancet Oncol (2020) 21(1):44–59. 10.1016/s1470-2045(19)30689-8 31786121

[65] Schmid P Adams S Rugo HS Schneeweiss A Barrios CH Iwata H. Atezolizumab and Nab-Paclitaxel in Advanced Triple-Negative Breast Cancer. N Engl J Med (2018) 379(22):2108–21. 10.1056/NEJMoa1809615 30345906

