# Supplementary figures and images for: PD-L1-Mediated Immunosuppression in Oral Squamous Cell Carcinoma: Relationship With Macrophage Infiltration and Epithelial to Mesenchymal Transition Markers
Source: Front Immunol. 2021 Sep 6;12:693881. doi: 10.3389/fimmu.2021.693881 (PMC8450501; doi:10.3389/fimmu.2021.693881)

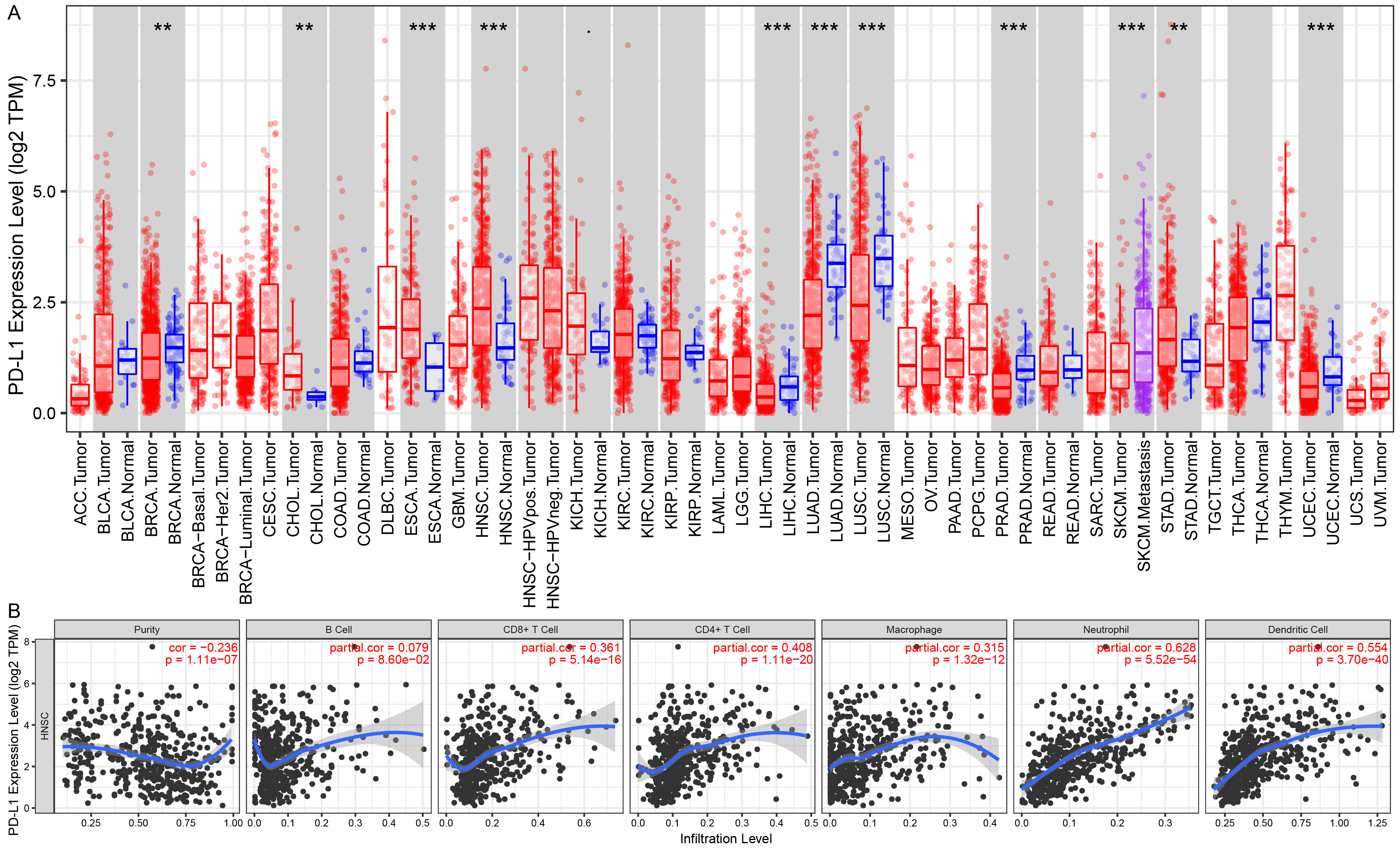

Supplement: Supplementary Figure 1 — PD-L1 expression level in various cancers. (A) PD-L1 expression level in various cancers was analyzed by box-plot. (B) Correlation between PD-L1 and immune infiltrates in HNSCC. [file Image_1.jpeg]

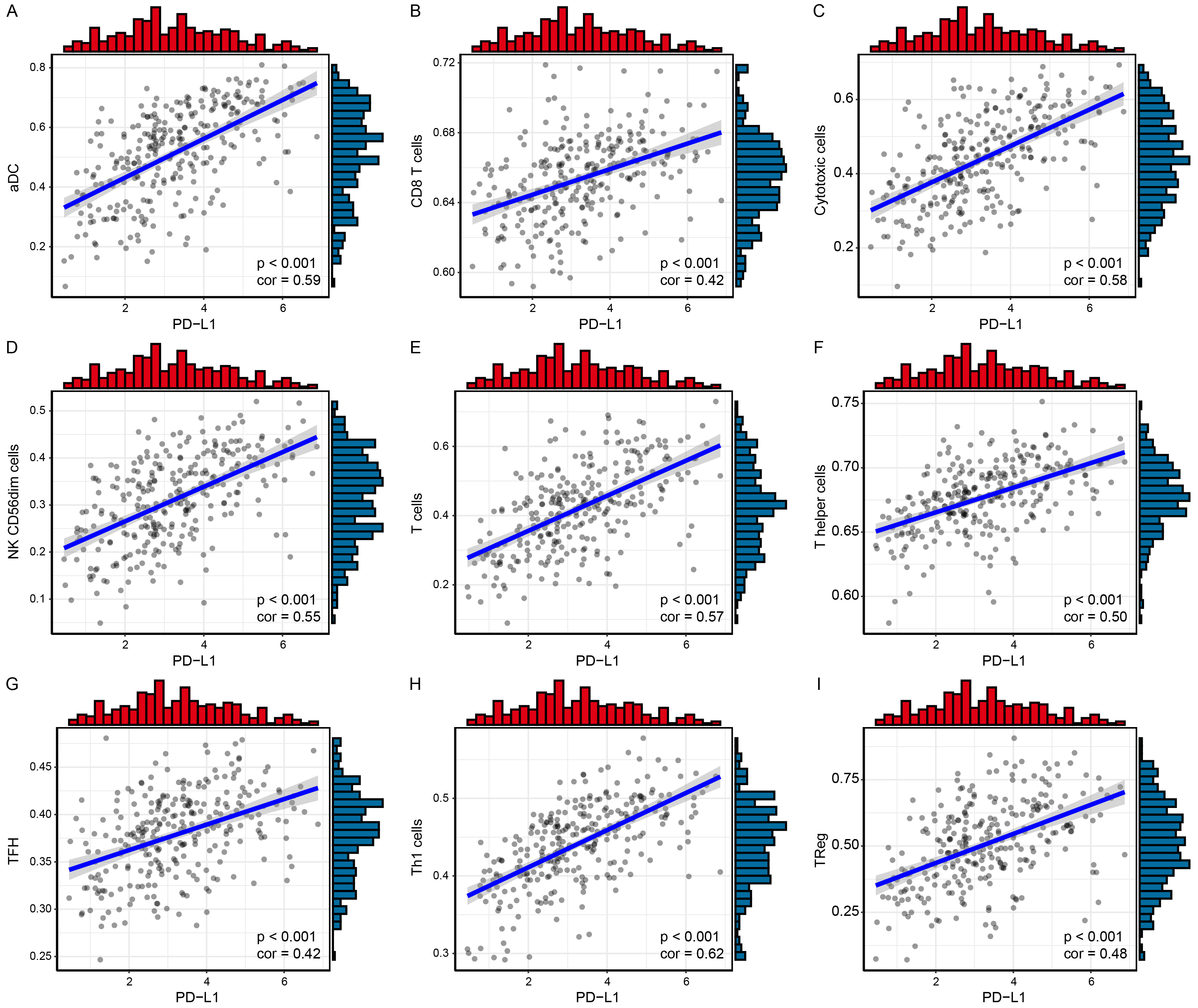

Supplement: Supplementary Figure 2 — Scatter diagram of the correlation between PD-L1 expression and immune cell infiltration in OSCC. [file Image_2.jpeg]

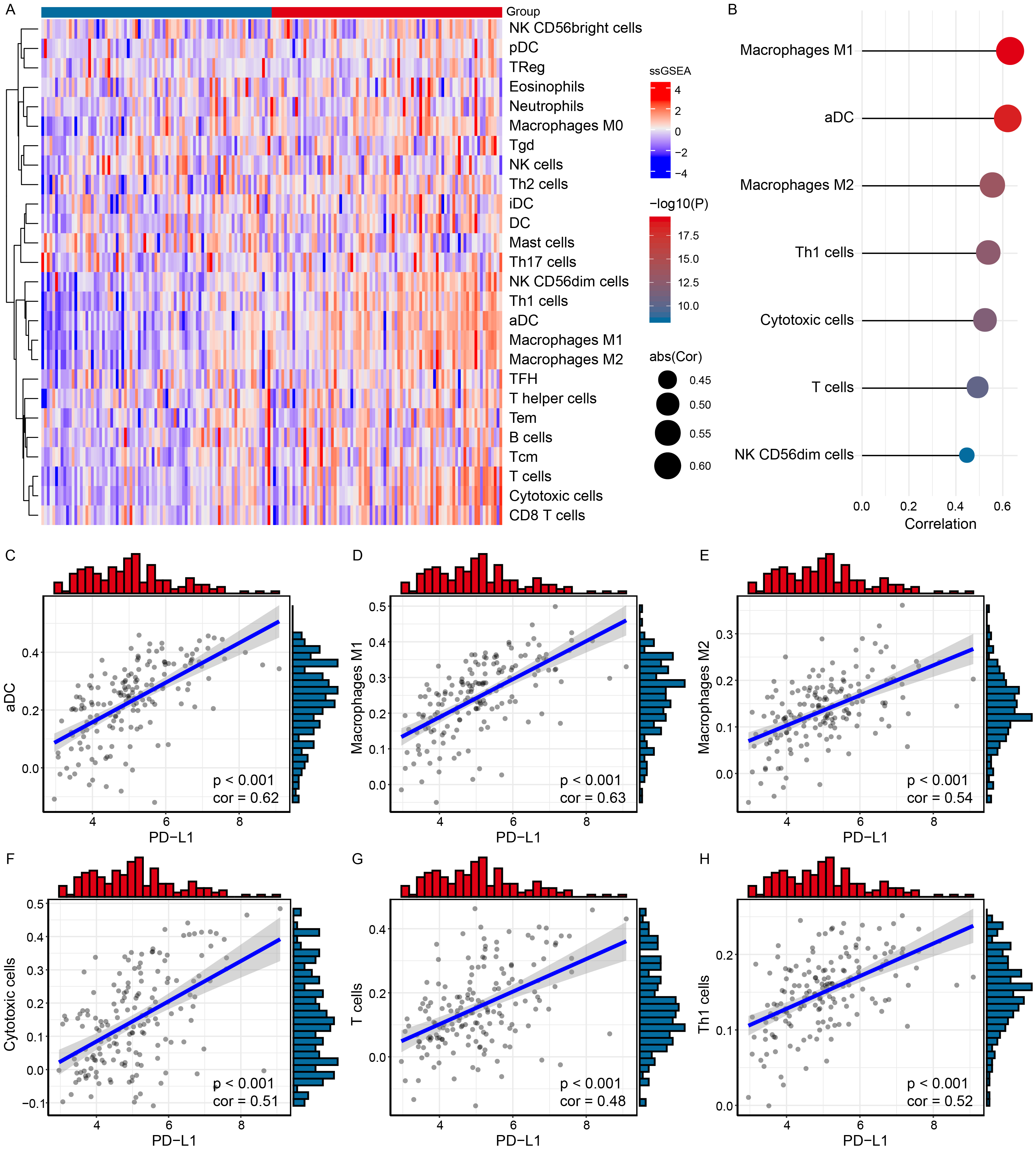

Supplement: Supplementary Figure 3 — Relationship between PD-L1 and immune infiltrates in the GEO validation set. (A) Heat map of OSCC samples by using the ssGSEA scores from 26 immune cell types. (B) PD-L1 significantly associated with Immune cells. Scatter plot showing correlation between PD-L1 and various immune cells, including aDC (C), M1 macrophages (D), M2 macrophages (E), Cytotoxic cells (F), T cells (G) and Th1 cells (H). [file Image_3.jpeg]
